# Editorial: Emerging Roles of Circular RNAs in the Tumor: Functions and Potential Applications

**DOI:** 10.3389/fcell.2022.846926

**Published:** 2022-01-31

**Authors:** Shanchun Guo, Lan Huang, Mingli Liu

**Affiliations:** ^1^ Department of Chemistry, Xavier University, New Orleans, LA, United States; ^2^ Biotherapy Center, the First Affiliated Hospital of Zhengzhou University, Zhengzhou, China; ^3^ Department of Microbiology, Biochemistry and Immunology, Morehouse School of Medicine, Atlanta, GA, United States

**Keywords:** circular RNAs, tumor, signaling pathways, diagnostics, therapeutics

Circular RNA (circRNA), which was firstly described in mammals in 1991, has undergone an explosion in studies on its biology resulting in the common understanding of the molecule’s role as important players in physiology and pathology/disease developments (Kristensen et al., 2018; Chen and Lu, 2021). CircRNAs are single-stranded RNAs produced by back splicing of exons and/or intron sequences of primary RNA transcripts. Compared to traditional linear RNAs formed by forward splicing, a 3′-5′ covalently closed ring within the circRNAs keeps them stable even without posttranscriptional modification such as the addition of 5′-cap and 3′-poly (A) tails.

CircRNAs are classified as four groups based on biogenesis and locations. Exonic circRNAs (EcircRNAs) are produced by back splicing from exon sequence and are predominantly localized in the cytoplasm. Exon-intro circRNAs (EIciRNAs) are circulized with intronic sequences retained between the back-splicing-exons and are localized in the nucleus. Intronic circRNAs (ciRNAs) from intronic lariat RNA precursors are localized in the nucleus as well, while mitochondria-encoded circRNAs (mecciRNAs) are distributed in the mitochondria and the cytoplasm (Chen et al., 2021). CircRNAs have diverse functions including 1) inhibiting miRNA activities by sponging miRNAs, 2) initiating RNA polymerase II (Pol II) to regulate transcription, 3) acting as a protein scaffold and a protein sponge, and 4) encoding proteins by serving as translation templates (Chen et al., 2021).

CircRNAs have been accepted as the specific regulators in gene expression rather than erroneous noise during gene transcription. Deregulation of circRNAs may influence proliferative signaling, epithelial-to-mesenchymal transition (EMT), angiogenesis, apoptosis and drug resistance, therefore may have a causative role in cancer development (Kristensen et al., 2018). So far, aberrant expressions of circRNAs have been identified in diverse cancer types and tissues, including basal cell carcinoma (BCC), bladder cancer, breast cancer, ovarian cancer, oral squamous cell carcinomas, esophageal squamous cell carcinoma (ESCC), gastric cancer, colorectal carcinoma (CRC), hepatocellular carcinoma (HCC), lung cancer, cutaneous squamous cell carcinoma, osteosarcoma, glioma, and hematological malignancies (Kristensen et al., 2018). The participation of circRNAs in cancer stemness (Su et al., 2019; Lagunas-Rangel, 2020) and tumor-associated immunology (Tang et al., 2020; Duan et al., 2021) further provided insight into their widespread roles in cancer. Therefore, circRNAs can be used as molecular markers and potential therapeutic targets for the early diagnosis and treatment of tumors. Activating and/or blocking the expression of circRNAs will effectively regulate the growth process of tumors and will provide a new therapeutic strategy.

The research topic “Emerging Roles of Circular RNAs in the Tumor: Functions and Potential Applications” focuses on circRNAs’ role and molecular signaling pathways by which they regulate tumor development. We have received 39 manuscripts contributed by 184 authors from all over the world and have selected 24 articles from multiple fields in cancer research that were ultimately published.

Five reviews/mini-reviews were collected in this research topic. The one from Nisar et al. systemically reviewed circRNAs’ biogenesis, properties, and characterization. The authors summarized circRNAs’ detection methods, overviewed their roles as diagnostic and prognostic markers in both solid tumors and hematological malignancies and then discussed their molecular targets. The other two reviews focused on specific cancers, prostate cancer, and thyroid cancer (TC). The one from Fang et al. reviewed circRNAs in the prostate cancer. The authors first overviewed the general features of circRNAs (resistance to exonuclease degradation, selective enrichment within exosomes, cell lineage- and tissue-specific features, and easy detection by high throughput RNA sequencing or multiplex qPCR in one single reaction), followed by discussion of the potential of circRNAs as diagnostic and prognostic biomarkers for prostate cancer. The other review from Xia et al. outlined the characteristics, expression, and function of circRNAs in TC, the highest incidence among endocrine malignancies. CircRNAs, as regulatory molecules, potentially serve as diagnostic, prognostic, and therapeutic tools in TC. Acquired comprehensive understanding of circRNAs leads to new therapies based on circRNAs in treating and preventing TC. Seimiya et al. reviewed the role of exosomal circRNAs in cancer. They discussed the biological functions of exosomal circRNAs and their contribution to cancer progression. The potential clinical applications of exosome circRNAs as biomarkers and therapeutic targets for cancer were also summarized in this review. Jiang et al. focused on the function of the most well-known circRNAs, cerebellar degeneration-related protein 1 antisense RNA (CDR1as) in the development of various cancers such as colorectal cancer, cholangiocarcinoma and osteosarcoma. CDR1as contains over 70 miR-7 (a notable tumor suppressor) binding sites and can regulate gene expression by sponging miR-7, which contributes to tumor growth, metastasis, and chemoresistance. Besides miR-7, CDR1as exerts its role *via* multiple signaling pathways, for instance, stimulating tumor growth through miR-1270, miR-219a-5p, and miR-135p; promoting cancer metastasis *via* IGF2BP3, miR-1270, miR-219a-5p, miR-876-5p, miR-641, and miR-1299; and enhancing resistance to chemotherapy by miR-1270.

Nineteen original research articles are collected in this research topic. Two studies focused on prostate cancers. By screening a circRNA microarray assay, Deng et al. found that circ_0088233 was upregulated in prostate cancer tissues compared to adjacent normal tissues and these findings were verified in 46 pairs of prostate cancer and adjacent normal tissues. Levels of circ_0088233 correlated with TNM stages. Reduced expression of circ_0088233 inhibited cell proliferation and migration/invasion as well as induced G1 phase arrest and apoptosis. Furthermore, has-miR-185-3p was identified as the downstream target of circ_0088233. Their results implicate that circ_0088233 may function as an oncogene and play an oncogenic role by sponging hsa-miR-185-3p, therefore this represents a potential therapeutic target for prostate cancer. Greene et al. detected circRNA profiling using a panel of prostate cell lines comprising normal, benign, and malignant cells. They identified three circRNAs, hsa_circ_0021652, hsa_circ_0000288, and hsa_circ_0021647, that are associated with *Caprin1* gene, an established survival and chemical resistance marker for prostate cancer.

In two studies, Hippo/Yes-associated protein (YAP) signaling cascades were investigated for their relationship with upstream non-coding RNAs. Circ_100395 functions as a tumor suppressor in non-small cell lung carcinoma (NSCLC). Zhang et al. isolated circ_100395 from adipose-derived mesenchymal stem cells (AMSC), which can increase LATS2 expression by sponging miR-141-3p to regulate the Hippo/YAP signaling cascade, therefore inhibiting NSCLC malignant transformation. Interestingly, Hippo/YAP signaling pathways also contribute to triple-negative breast cancer (TNBC) progression regulated by non-coding RNA LncRNA GHET1. Wang and Liu assessed the specific impacts and mechanisms of LncRNA GHET1 in the development of TNBC. Under hypoxia conditions, they found that knockdown of LncRNA GHET1 significantly decreased proliferation and invasion of TNBC cells, in parallel, accompanied by decreased glucose consumption and lactate production. Mechanically, knockdown of LncRNA GHET1 increased the phosphorylation levels of LATS1 and YAP, consequently, to retain YAP within the cytoplasm.

Under this research topic, the roles of circRNAs were also investigated in other solid tumors, including breast, ovarian cancer, cervical carcinoma, hepatic carcinoma (HCC), glioma, and papillary thyroid carcinoma (PTC). 1) The data from Karedath et al. suggested that CircNFATC3 is a functionally relevant circular RNA in breast and ovarian cancers. The knockdown of circNFATC3 induces a reduction in cell proliferation, invasion, and corresponding oxidative phosphorylation in breast and ovarian cancer cells. 2) The work from Sun et al. showed that circAMOTL1(hsa_circ_0004214) was highly expressed in cervical carcinoma patients and cervical carcinoma cell lines. Downregulation of circAMOTL1 could restrain malignant phenotypes, inactivation of AKT signaling, and repression of EMT phenotypes in cervical carcinomas. Mechanically, circAMOTL1 functions as a miR-526b sponge to upregulate the expression of salt-inducible kinase 2 (SIK2) and subsequently increases malignant phenotypes of cervical carcinoma cells. 3) Screening by quantitative reverse transcription-polymerase chain reaction (qRT-PCR) studies on 50 patients with HCC and 50 healthy individuals, Lin et al. reported that circ_0072088 was highly expressed in patients with HCC, while its increased expression indicated unfavorable prognosis for HCC patients. Furthermore, they found that circ_0072088 was mainly secreted into plasma by HCC cells through exosomes, whereas circ_0072088 suppresses migration and invasion of HCC through degradation of miR-375 and upregulation of MMP-16. 4) Studies from Li et al. showed that circGLIS3 (hsa_circ_0002874) play an important role in glioma. Their functional experiments showed that the upregulation of circGLIS3 in glioma promoted glioma cell migration and invasion and exhibited aggressive characteristics in tumor-bearing mice, while their mechanical experiments uncovered that circGLIS3 could activate Ezrin T568 phosphorylation. In addition, circGLIS3, secreted by glioma cells and enriched in exosomes, affected tumor environments through induction of angiogenesis. 5) By using GSE93522 dataset *via* the GEO2R online tools, Lou et al. first screened out 14 candidate circRNAs that differentially expressed between normal/benign thyroid tissues or PTC. After performing miRNA co-prediction by the combination of Cancer-Specific CircRNA Databases (CSCD) and Circular RNA interactome (CRI) databases, they constructed a potential circRNA-miRNA sub-network, consisting of 9 circRNAs and 21 miRNAs. Following subsequent determination of the expression and prognostic values of these miRNAs by starBase, the two miRNAs, miR-605-5p, and miR-876-3p, were identified as key miRNAs in PTC. Subsequently, CTNNB1 and CCND1 were found to be the two most likely targets of miR-876-3p. Then they concluded that hsa_circ_0088494-miR-876-3p-CTNNB1/CCND1 axis linked to carcinogenesis and progression of PTC, which provide promising therapeutic targets for PTC.

In addition to solid tumors, this research topic also includes the contribution of circRNAs to hematological malignancy. Molin et al. in Italy used the CirCormPara software to annotate and quantify circRNAs in RNA-seq data comprising of 19 Juvenile myelomonocytic leukemia (JMML) and 3 healthy donors (HD). They identified 119 circRNAs dysregulated in JMML and 59 genes showing an imbalance between circRNAs and linear RNA products. Varied circRNA expression profiles among molecular subgroups of JMML were also identified. After validation of a set of dysregulated circRNAs in an independent cohort of JMML patients, they confirmed the downregulation of circOXNAD1 and circATM, and a significant upregulation of circLYN, circAFF2, and circMCTP1 in JMML patients. The study model they used predicted the existing interactions with miRNAs and dysregulated circRNAs in regulatory networks.

In conclusion, this research topic highlights multiple roles and contribution of non-coding RNAs, in particular, circRNAs, in a variety of human tumors. However, many aspects of circRNA research still needs to be explored, for example, how is the circRNA expression related to other molecular changes frequently observed in cancers such as *KRAS*, *c-myc*, and *p53*? Finally, there is no doubt that circRNA is a promising candidate as biomarker for cancer and therapeutic target in cancer therapy.
